# Perspective: One-Cell and Cleavage-Stage Mouse Embryos Thrive in Hyperosmotic Oviductal Fluid Through Expression of a Glycine Neurotransmitter Transporter and a Glycine-Gated Chloride Channel: Clinical and Transgenerational Implications

**DOI:** 10.3389/fphys.2020.613840

**Published:** 2020-12-21

**Authors:** Lon J. Van Winkle

**Affiliations:** ^1^ Department of Biochemistry, Midwestern University, Downers Grove, IL, United States; ^2^ Department of Medical Humanities, Rocky Vista University, Parker, CO, United States

**Keywords:** ART, embryo, epigenetic modifications, glycine, GLYT1, GLRA4, metabolic syndrome, oviductal fluid

## Abstract

The osmolality of mouse oviductal fluid ranges from about 300 mOsmol/kg in the ampulla 0–3 h post coitus (h p.c.) to more than 350 mOsmol/kg in the isthmus 34–36 h p.c. Thus, it has been surprising to find that development of one-cell and cleavage-stage mouse embryos arrests *in vitro* in media exceeding 300 mOsmol/kg, and they develop best in unphysiological, hypotonic media. The glycine concentration in oviductal fluid can, however, rescue development in hypertonic media, so physiological conditions *in vivo* and *in vitro* likely work together to foster embryo well-being. Glycine acts on one-cell and cleavage-stage mouse embryos through the glycine-gated chloride channel, GLRA4, and uptake *via* the glycine neurotransmitter transporter, GLYT1. Since these processes lead to further signaling in neurons, the presence and function of such signaling in preimplantation embryos also should be investigated. The more we know about the interactions of physiological processes and conditions *in vivo*, the better we would be able to reproduce them *in vitro*. Such improvements in assisted reproductive technology (ART) could improve patient outcomes for IVF and potentially help prevent unwanted developmental abnormalities in early embryos, which might include undesirable epigenetic DNA and histone modifications. These epigenetic modifications may lead to transgenerational adult disorders such as metabolic syndrome and related conditions.

## Introduction

One-cell and cleavage-stage mouse embryos employ several biomembrane transport processes to regulate their cellular volumes (reviewed in Baltz, 2001 and Van Winkle, 2001). Perhaps most surprising of these processes is the neurotransmitter transporter, GLYT1, which functions to accumulate glycine as an osmolyte in embryos (Steeves et al., 2003). This transporter has been thoroughly characterized in early embryos (Hobbs and Kaye, 1985; Van Winkle et al., 1988), and it has been definitively identified as GLYT1 (Van Winkle and Campione, 1996). Initially, glycine was shown to protect preimplantation embryos from the adverse effects of oviductal fluid-like medium, because it serves as an intracellular osmolyte to resist cellular shrinkage in such hypertonic media (Van Winkle et al., 1990). Since then, organic osmolyte-reversed detrimental effects of hypertonic media on preimplantation development have been observed for several amino acids beginning at a medium osmolality of about 300 mOsmol/kg (Baltz, 2001; Van Winkle, 2001).

More recently, the glycine-gated chloride channel, GLRA4, was also shown to facilitate development of fertilized mouse eggs into blastocysts (Nishizono et al., 2020). This channel appears to catalyze chloride uptake by embryonal cells in the presence of glycine. Hence, extracellular glycine appears to promote preimplantation mouse embryo development as a ligand as well as an intracellular osmolyte. Glycine likely increases in importance as an intracellular osmolyte as oviductal fluid becomes more hypertonic.

## Attempts to Measure Oviductal Fluid Osmolality may Have Exposed Glra4 Activity

Because too little fluid is present in the mouse oviduct to measure its osmolality with an osmometer (i.e., only picoliters of fluid; Borland et al., 1977), one-cell embryos have been used as “osmometers” to attempt to estimate the osmolality of oviductal fluid (Collins and Baltz, 1999). It was concluded that this fluid has an osmolality of about 290–300 mOsmol/kg based, in part, on the osmolality at which zygotes did not shrink or swell beginning 1.8 min after they were placed in media of various osmolalities between 200 and 400 mOsmol/kg. The same approach for two-cell embryos yielded a similar conclusion (Collins and Baltz, 1999; Pogorelova et al., 2011). Not considered, however, were initial changes in zygote and embryo volume from when they were in oviductal fluid (e.g., Figure 2 in Collins and Baltz, 1999) to when volumes were measured in media 1.8 min later (Figure 3 in Collins and Baltz, 1999). For these reasons, we reassessed these interesting data to include volume changes in fertilized eggs over their first 1.8 min in media of various osmolalities.

**Figure 1:**
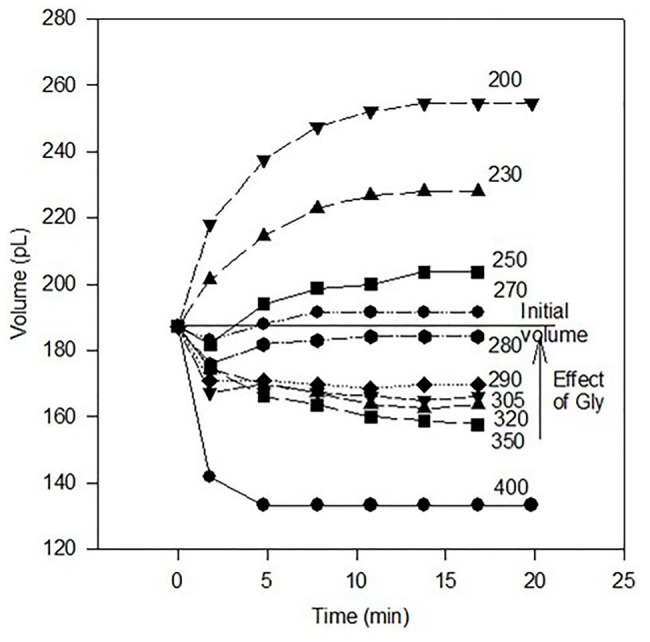
Volume of mouse zygotes as a function of time after removal from oviducts in medium of the indicated osmolality (mOsmol/kg). Data are from Collins and Baltz (1999, Figure 3) re-illustrated to show the volumes of embryos relative to their initial volume in oviductal fluid (Figure 2; Collins and Baltz, 1999). Embryos in each medium either decreased or increased in volume at 1.8 min relative to their initial volumes at 0.0 min in oviductal fluid (*p* < 0.05, ANOVA). Subsequently, all zygotes either increased or decreased in volume as shown (*p* < 0.01) except for those in medium of 290 or 305 mOsmol/kg (no statistically significant change after the initial volume decrease). Mean volumes are indicated by each symbol, and the standard errors of the mean volumes are within the limits of the symbols (*n* = 19–26 for each mean volume except for the initial volume at *t* = 0.0 min where *n* = 153). Also indicated is the rescue of zygote volume as well as development owing to addition of 1.0 mM glycine to 350 mOsmol/kg medium (see text).

When compared to their volumes in oviductal fluid, zygotes shrink initially when placed in culture media of 250 mOsmol/kg or higher (Figure 1). Together, these data might be taken erroneously to mean that the osmolality of oviductal fluid is about 275 mOsmol/kg i.e., the osmolality at which zygote volume at equilibrium is equal to zygote volume in oviductal fluid. However, we attribute the initial volume decrease even in somewhat hypotonic conditions to the absence of glycine in the media.

In the absence of glycine, glycine-gated chloride channels would close. Consequently, the chloride influx they catalyze would cease, and one-cell embryos would shrink until compensatory transport processes could occur (e.g., see 250–280 mOsmol/kg data in Figure 1). Perhaps more importantly, when glycine is present in the medium, it protects against one-cell embryo shrinkage even in medium of 350 mOsmol/kg (Steeves et al., 2003). Hence, most of the data in Figure 1, and data reported elsewhere (Collins and Baltz, 1999; Pogorelova et al., 2011), were collected under non-physiological conditions (in particular, in the absence of extracellular glycine in the medium).

## Direct Measurement of the Major Osmolytes in Oviductal Fluids

The osmolality of oviductal fluids can be calculated by adding the molar concentrations of its major inorganic osmolytes (Borland et al., 1977; Van Winkle et al., 1990). Such calculations are likely underestimates, since they include Na^+^, Cl^−^, K^+^, Mg^2+^, and Ca^2+^ but not organic osmolyte. They also include HCO_3_^−^, which is assumed to be equal to the excess positive charge of these positive and negative ions (i.e., 13–25 mM; Borland et al., 1977). These are likely underestimates of the HCO_3_^−^ concentrations, since the HCO_3_^−^ concentration is about 33 mM in rabbit oviductal fluid (David et al., 1981). Nevertheless, the calculations reveal the extent to which the fluids are hypertonic.

According to these calculations, the osmolarity of oviductal fluid increases from 309 mM in the ampulla at 0–3 h post coitus (h p.c.) to 350 mM in the ampulla at 11–13 h p.c. and 368 mM in the isthmus at 34–36 h p.c. as development proceeds from the one-cell to the two-cell stage (Borland et al., 1977). Because oviductal fluids are biological, these numbers should be reduced by about 15 mM owing to incomplete dissociation of the inorganic salts (Baltz, 2001). Nevertheless, one-cell and cleavage-stage mouse embryos need to survive and continue to develop in oviductal fluid that has an osmolality sometimes exceeding 350 mOsmol/kg.

Moreover, such changes in the composition of reproductive tract fluid over time and along the tract should help us better to produce physiologically more normal conditions for preimplantation mouse embryo development *in vitro* (Morris et al., 2020). Other attempts, to measure the osmolality of mouse oviductal fluid more directly using a microsmometer, used samples from the whole oviduct after it was torn open at the one-cell stage of development (Fiorenza et al., 2004). The resultant value of 302 ± 4 mOsmol/kg is within the range of values calculated above from the ionic composition of oviductal fluid, but it does not reflect time or position changes in oviductal fluid osmolality as development proceeds. It is likely important to use the mouse model, including temporal and positional changes in oviductal fluid composition along the reproductive tract, to help recreate the *in vivo* environment of human as well as mouse embryos *in vitro*. Children conceived by assisted reproductive technology (ART) using non-physiological culture media have increased risk of preterm birth, low and high birth weight, birth defects, and metabolic dysfunction (See clinical implications below).

## The Glycine Neurotransmitter Transporter, Glyt1, Accumulates the Osmolyte, Glycine

Glycine in the culture medium completely rescues the ability of one-cell embryos to develop to the four-cell stage up to an osmolarity of at least 350 mOsmol/kg (Figure 1 and reviewed in Baltz and Tartia, 2010). Concomitantly, the ability of GLYT1 to accumulate glycine in one- and two-cell embryos increases linearly from 250 to about 370 mOsmol/kg, and these embryos also accumulate glycine *in vivo*. Moreover, the inhibitor of GLYT1, ORG23798, blocks glycine rescue of embryo development in medium with osmolality of 310 mOsmol/kg, but the inhibitor has no effect on development of one-cell embryos in somewhat hypotonic medium (Steeves et al., 2003). Hence, glycine uptake by GLYT1 is required for one-cell embryo development to the 4–8-cell stage in hypertonic media. Osmolalities sometimes in excess of 350 mOsmol/kg are likely present in the oviduct during this developmental period (see above).

## The Glycine-Gated Chloride Channel, Glra4, Fosters Chloride Influx

When cultured in somewhat hypotonic, glycine-containing medium, fertilized mouse eggs are inhibited in their progression to the four-cell stage and arrest as morula when cultured with the glycine-receptor inhibitors, strychnine and PMBA (Nishizono et al., 2020). These receptors are glycine-gated chloride channels. GLRA4 is expressed in fertilized mouse oocytes and early embryos, while GLRA1 is expressed in fertilized bovine oocytes, and GLRA2 is expressed in human zygotes (Nishizono et al., 2020). Finally, *Glra4*-knockout slowed preimplantation development of fertilized mouse eggs, and the resultant blastocysts had fewer cells. Litter sizes were also smaller in *Glra4*-knockout mice (Nishizono et al., 2020). Thus, glycine-stimulated chloride influx likely fosters mouse one-cell and cleavage-stage development *in vivo*. Moreover, glycine-gated chloride channel activity is needed for preimplantation mouse embryo development even when glycine uptake is not needed as an osmolyte to counteract hyperosmotic conditions *in vitro*.

## Are Glycine and Chloride Transport as Osmolytes Their Full Function or do They Also Produce Signaling?

Amino acid transport is frequently associated with signaling by the amino acid or even the transport process itself. For example, transport of leucine by system B^0,+^ in blastocysts initiates the mTOR signaling needed for development of trophoblast motility and penetration of the uterine epithelium. Leucine uptake by other processes in blastocysts does not lead to trophoblast motility, so system B^0,+^ either selectively directs leucine to mTOR, or another signaling process is also initiated during leucine transport by system B^0,+^ (reviewed in Van Winkle and Ryznar, 2018, 2019a,b). Similarly, threonine transport into mammalian embryonic stem cells likely triggers signaling through the transport process itself, as well as signaling by intracellular threonine and its metabolites (Van Winkle and Ryznar, 2019a,b). Since GLYT1 and GLRA4 both function for signaling in neurons (Moss and Smart, 2001; Gabernet et al., 2004; Kopec et al., 2010), a similar phenomenon could occur in one-cell and cleavage stage embryos. This possibility warrants exploration in human as well as mouse embryos.

## Clinical and Transgenerational Implications

Human ART currently uses culture media that are known not to mimic physiological conditions as closely as possible. For example, these media have relatively low osmolalities (Baltz and Tartia, 2010), while the mean osmolality of human oviductal fluid equals 316.6 mOsmol/kg (SEM = 2.5 mOsmol/kg) as measured using an osmometer (Canha-Gouveia et al., 2020). Hence, it could become beneficial to create culture conditions for oocytes and preimplantation embryos *in vitro* that more nearly reflect conditions *in vivo*. Such conditions likely differ among species, since one-cell rat embryos develop better in media of 304 than 246 mOsmol/kg (Yang et al., 2004), whereas the reverse is true for the fertilized mouse eggs discussed above. The osmolality of oviductal fluid in the cycling rat was reported to be 287 ± 1 mOsmol/kg (Waring, 1976), but this osmolality has not, to our knowledge, been measured during pregnancy. To further complicate work to reproduce *in vivo* conditions *in vitro*, one-cell and cleavage-stage embryos from different strains of mice exhibit different thresholds of susceptibility to impaired development by hypertonic media, although glycine rescues development in all of the strains that have been tested (Suzuki et al., 1996; Hadi et al., 2005). Nevertheless, human as well as mouse zygotes express a glycine neurotransmitter transporter and a glycine-gated chloride channel (Baltz and Tartia, 2010; Nishizono et al., 2020), and these transport proteins are likely important to the development of early embryos of both species *in vivo*. Possibly because early human embryos develop *in vitro* in non-physiological conditions, children conceived by ART have increased risk of preterm birth, low and high birth weight, birth defects, and metabolic dysfunction (Feuer and Rinaudo, 2012).

For example, initial meta-analyses showed increased risks of cardiovascular disease, higher body fat composition, greater fasting blood glucose, and elevated blood pressure in children conceived through ART (Hart and Norman, 2013; Guo et al., 2017). At birth, these children were more likely to be preterm, smaller, and exhibit birth defects (Chang et al., 2020). Subsequently, such children exhibited increased fasting blood glucose, higher insulin levels, and greater HOMA-IR, but lower HDL ApoA (Cui et al., 2020). These unwanted characteristics appear to arise in mammals through alterations in development. If such changes are the result of epigenetic DNA and histone modifications, then the changes could be transgenerational (Ventura-Juncá et al., 2015; Van Winkle and Ryznar, 2018, 2019a,b; Choux et al., 2020). To address the risk of future disease for early mouse or human embryos developing in hypotonic vs. hypertonic medium plus or minus glycine, a two-by-two experiment would be relatively easy to design. Because the *in vivo* environment is much more complex, however, and involves growth factors and other physical and chemical conditions (e.g., Kaye, 1997), there are numerous dimensions to the media that should be tested in animal models of preimplantation embryo development before they are examined in humans.

## Conclusions

As calculated above, the osmolality of mouse oviductal fluid ranges from about 300 mOsmol/kg in the ampulla 0–3 h p.c. to more than 350 mOsmol/kg in the isthmus 34–36 h p.c. In contrast, one-cell and cleavage-stage mouse embryos develop best in unphysiological, hypotonic media *in vitro*, and their development arrests in media with osmolalities of 300 mOsmol/kg and above. Since the concentrations of glycine found in oviductal fluid rescue development in hypertonic media, physiological conditions *in vivo* and *in vitro* likely work together to foster embryo well-being. Glycine acts on one-cell and cleavage-stage mouse embryos through the glycine-gated chloride channel, GLRA4, and uptake *via* the glycine neurotransmitter transporter, GLYT1. Moreover, these processes lead to further signaling in neurons, so the presence and function of such signaling in preimplantation embryos also should be investigated. The more we know about the interactions of physiological processes and conditions *in vivo*, the better we would be able to reproduce them *in vitro*. These improvements in ART could improve patient outcomes for IVF and potentially help to prevent unwanted developmental abnormalities in early embryos, which might include undesirable epigenetic DNA and histone modifications. Undesirable epigenetic modifications may lead to transgenerational adult disorders such as metabolic syndrome and related conditions.

## Data Availability Statement

The original contributions presented in the study are included in the article/supplementary material, further inquiries can be directed to the corresponding author.

## Author Contributions

The author confirms being the sole contributor of this work and has approved it for publication.

### Conflict of Interest

The author declares that the research was conducted in the absence of any commercial or financial relationships that could be construed as a potential conflict of interest.
